# Motivational interviewing skills practice enhanced with artificial intelligence: ReadMI

**DOI:** 10.1186/s12909-024-05217-4

**Published:** 2024-03-05

**Authors:** Paul J. Hershberger, Yong Pei, Dean A. Bricker, Timothy N. Crawford, Ashutosh Shivakumar, Angie Castle, Katharine Conway, Raveendra Medaramitta, Maria Rechtin, Josephine F. Wilson

**Affiliations:** 1 Department of Family Medicine, Wright State University Boonshoft School of Medicine, Dayton, OH USA; 2 Department of Computer Science, College of Computing and Software Engineering, Kennesaw State University, Kennesaw, GA USA; 3 Department of Internal Medicine, Wright State University Boonshoft School of Medicine, Dayton, OH USA; 4 Department of Population and Public Health Sciences, Wright State University Boonshoft School of Medicine, Dayton, OH USA; 5 Department of Computer Science and Engineering, College of Engineering and Computer Science, Wright State University, Dayton, OH USA; 6 Boonshoft School of Medicine, Wright State University, Dayton, OH USA

**Keywords:** Motivational interviewing, Artificial intelligence, Medical education, Patient engagement, Feedback

## Abstract

**Background:**

Finding time in the medical curriculum to focus on motivational interviewing (MI) training is a challenge in many medical schools. We developed a software-based training tool, “Real-time Assessment of Dialogue in Motivational Interviewing” (ReadMI), that aims to advance the skill acquisition of medical students as they learn the MI approach. This human-artificial intelligence teaming may help reduce the cognitive load on a training facilitator.

**Methods:**

During their Family Medicine clerkship, 125 third-year medical students were scheduled in pairs to participate in a 90-minute MI training session, with each student doing two role-plays as the physician. Intervention group students received both facilitator feedback and ReadMI metrics after their first role-play, while control group students received only facilitator feedback.

**Results:**

While students in both conditions improved their MI approach from the first to the second role-play, those in the intervention condition used significantly more open-ended questions, fewer closed-ended questions, and had a higher ratio of open to closed questions.

**Conclusion:**

MI skills practice can be gained with a relatively small investment of student time, and artificial intelligence can be utilized both for the measurement of MI skill acquisition and as an instructional aid.

## Background

The management of chronic illness is a major public health and medical challenge internationally, a challenge that high-income or high healthcare-spending countries have not been able to avoid. For example, in the United States, 60% of adults have at least one chronic condition and over 40% have more than one [1–2]. Despite spending more on healthcare than any other country in the world, the prevalence of diabetes in the adult population in the United States is over 50% higher than in other high-income countries, and the obesity rate is over 70% higher [3]. The chronic disease burden is a global challenge as noncommunicable diseases (NCDs) are responsible for approximately three-quarters of all deaths internationally each year [4]. In any country, the effective management of chronic illnesses depends more on the behavior of the patient than what the medical professional can contribute [5–7], so patients’ active engagement in their own care is crucial. When management plans are tailored to the patient’s goals and priorities [8–10], higher levels of patient activation are associated with better adherence and improved health outcomes for individuals with chronic conditions [11–14]. Accordingly, improvement in chronic disease management will necessarily involve more effective attention to patient engagement and activation, consistent with the World Health Organization’s framework for “people-centered care.” [15].

Motivational interviewing (MI) is an evidence-based, brief interventional approach that has been demonstrated to be highly effective in increasing patient activation [16–17]. MI is a patient-focused conversation between the clinician and the patient that reinforces the patient’s motivation to make positive changes in any targeted health behavior through exploration of the patient’s normal and natural ambivalences [18–21]. Clinicians focus on being empathetic, nonjudgmental, and compassionately curious to help patients express their perspective on behavioral change and take responsibility for their choices. While MI can be effectively taught to medical students, residents, and practicing physicians [22–23], this patient-centered approach tends to be underutilized due to limited and inadequate training [24–25]. Therefore, an important gap in how chronic disease management is addressed is inadequate attention in medical education to training physicians in an evidence-based patient engagement approach such as motivational interviewing.

In spite of the increasing emphasis on patient-centeredness in medical education, the MI approach can be difficult to teach as a natural inclination of many physicians and other healthcare clinicians is to take a directive role by educating and instructing the patient with steps to improve health [26–27]. However, knowledge itself, if not combined with substantial motivation, rarely leads to behavior change. MI involves eliciting from the patient their own reasons for making a change, rather than the clinician debating with and/or trying to convince the patient to change (i.e., the spirit of motivational interviewing) [28]. During MI training, providers learn to talk less, listen more, use reflective statements, and ask open-ended questions– critical skills in the MI approach. Real-time assessment of these skills is advantageous in this learning process, as timely and targeted feedback is central to professional development [29].

### “ReadMI” training tool

We have developed a software-based MI skills-measuring program, “Real-time Assessment of Dialogue in Motivational Interviewing” (ReadMI), that aims to advance the skill acquisition of medical students as they learn the MI approach to address the behaviors that are crucial in effective chronic disease management [30–31]. ReadMI makes use of deep-learning-based speech recognition and natural language processing, implemented through mobile-cloud computing technologies to produce a spectrum of MI-relevant metrics. This human-artificial intelligence (AI) teaming helps reduce the cognitive load on a training facilitator, such that while ReadMI produces feedback on specific communication skills, the facilitator can give more attention to the overall quality and content of the conversation. The metrics produced by ReadMI include: provider versus patient talking time, the number of open-ended and closed-ended questions used, the use of reflective statements, the use of 0–10 scales (for rating importance, readiness, confidence, etc.), and a ratio of the number of reflections and questions. Producing immediate feedback on these MI-relevant metrics minimizes the need for time-consuming reviews of recorded training sessions which has typically been done in MI training, and helps eliminate delay in feedback. Additionally, a tool such as ReadMI can help make necessary follow-up coaching and feedback sessions more realistic as feedback is less cumbersome to produce [32]. We previously demonstrated that ReadMI had similar accuracy as human raters in identifying the types of questions and statements spoken by MI trainees [31]. The purpose of this study was to employ ReadMI as a means to both assess skill development and evaluate the extent to which its use affected the acquisition of MI-relevant skills. We hypothesized that students receiving ReadMI metrics as part of their training feedback would improve in MI-relevant conversational skills more than students not receiving ReadMI metrics in their training feedback.

## Methods

### Participants

The first 20 months of our medical school curriculum constitute the pre-clinical curriculum, and students then begin their required clinical rotations. During this “third year,” medical students (*N* = 125) doing their required Family Medicine Clerkship participated in the study. There were six clerkship rotations during the academic year (June 2020– March 2021), with each rotation including approximately 21 students. The Wright State University Institutional Review Board (IRB) determined that the study was exempt from human subjects review as it involved research on the effectiveness of our comparison of instructional techniques (IRB #06719, June 18, 2019). Before the educational activity, the informed consent process consisted of providing medical students with a clear description of the research being conducted as part of the evaluation of the educational experience.

### MI training experience

**Fig. 1:**
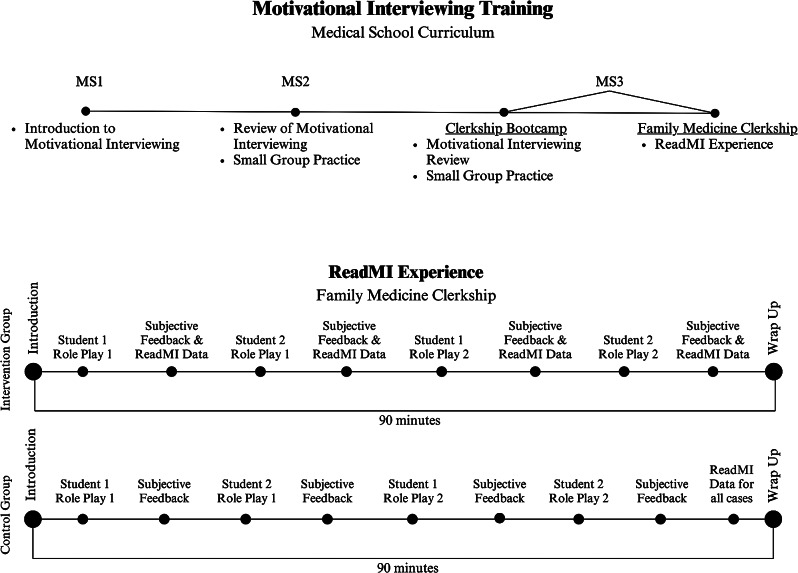
Motivational interviewing training progression

MI is introduced to students at our medical school early in their first year, reviewed during the second year, and then given additional attention in the preparatory “bootcamp” prior to students beginning their clerkships. During their Family Medicine clerkship, third-year medical students are scheduled in pairs to participate in a 90-minute MI training session with an MI facilitator. Figure 1 illustrates this progression of training, as well as the steps in the ReadMI experience for the intervention and control groups, respectively. Students complete an MI knowledge test prior to this training [33]. The training itself involves four role-plays, involving common Family Medicine scenarios, in which each student plays the role of a patient in two cases and the role of the physician in the other two cases. The MI training sessions are conducted virtually, using the Jitsi video conferencing system [34], and make use of the ReadMI training tool.

### Procedure

The study utilized a group rather than an individual randomization process. Prior to the academic year, the six Family Medicine clerkship cohorts were randomly assigned to an Intervention condition or a Control condition, so that all students in a given rotation were in the same condition. Students in both conditions received subjective feedback from the facilitators after each role-play, feedback that would typically make reference to relevant skills such as the use of open-ended questions, reflective statements, or the use of 0–10 scales. However, in the Intervention condition, feedback also included the specific numerical metrics produced by ReadMI, so that knowledge of the ReadMI metrics from the first role-play could be incorporated into the subsequent role-play. The ReadMI metrics were: provider talking time (percentage), patient talking time (percentage), number of open-ended questions, number of closed-ended questions, number of reflective statements, the number of times 0–10 scales (for rating importance, readiness, confidence, etc.) were used, and a ratio of the number of reflections to questions. In the Control condition, ReadMI metrics were provided to the students only after all four role-plays had been completed, so that these metrics would not influence performance in the second set of role-plays.

There were two facilitators for this educational activity, a psychologist and a licensed professional clinical counselor. Only one facilitator worked with each pair of students, although both facilitators participated in both the Control and Intervention conditions with each having five pairs of students for each rotation. Facilitator/student pairings were done based upon the schedules of the facilitators and students involved. There were approximately five minutes of facilitator feedback for each role play, regardless of whether the students were in the Control or Intervention condition. The content of the feedback varied based upon the performance of the student in the “doctor” role for that role play and was not controlled or scripted. The facilitators aimed to highlight what they perceived the student had done well and what they could improve upon, both with respect to conversational skills and the quality/content of the interview.

A brief description of the four role-plays is included in Table 1. The order of the four role plays was randomized for each pair of students. The student volunteering to be the physician first was given the initial role-play in the randomized order for that pair. Both students were given a brief description of the case prior to the beginning of each role-play, respectively, and the student playing the role of the patient (or parent) was instructed to “fill in the blanks with what you think would be typical for patient based upon this description.” The facilitator served as a timekeeper for the role-plays and stopped each role-play after seven or eight minutes unless the student playing the role of the physician finished before that time.

**Table 1:** Description of content of four role plays

• 55-year-old individual with a history of obesity (BMI 34), hypertension, and pre-diabetes. Doesn’t want to get the influenza vaccine. A few years ago when he got the vaccine he got sick with vomiting and diarrhea a few days later; attributes this illness to the vaccine.
• 35-year-old smoker (smoking x 20 years) who uses smoking to relieve stress. Not particularly concerned about health problems that might develop in the future.
• 40-year-old with good job and decent marriage. Doesn’t drink during the week but drinks heavily on the weekends, to the extent that most weekends are just a blur. Spouse drinks some but not as much as patient. Drinking hasn’t interfered with work, does not get aggressive when intoxicated, and hasn’t had any DUIs (even though acknowledges some driving after drinking). “Just having a good time” on weekends and doesn’t agree with doctor that this is a problem.
• 60-year-old with numerous chronic medical problems (Type II diabetes, hypertension, coronary artery disease, obesity) for which many medications are taken. Frustrated with the number of medications and doesn’t always take them. Tends to be sedentary and does not have a healthy diet in spite of encouragement to do so.

### Assessment

The Motivational Interviewing Knowledge Test (MIKT) was completed by students prior to the MI practice sessions and was used to statistically control for MI knowledge in our analyses [31]. The MIKT contains 22 questions, and the number of questions correct was summed to create a score. Validity and reliability data on this instrument have not been published.

ReadMI has been found to analyze and categorize the type of question/statement for interview transcripts nearly as well as human raters; the intraclass correlation coefficient was 0.828 [31]. With respect to correlations between the conversational metrics produced by ReadMI, higher percentages of physician speaking time is significantly related to lower numbers of open-ended questions, fewer reflections, and less use of 0–10 scales [31]. ReadMI metrics served as data for comparing student interview performance from Role-Play #1 to Role-Play #2.

As part of this educational activity, students also wrote paragraphs reflecting on their experience with MI practice as a means to gain qualitative feedback on this educational activity.

### Data analysis

Means and standard deviations were calculated for continuous variables, and frequencies and percentages were calculated for categorical variables. To examine differences in the ReadMI metrics and MIKT scores between the intervention and control groups for each session and between sessions, independent and paired t-tests were conducted. Standardized mean differences (Cohen’s D) were calculated to estimate effect sizes. Effect sizes of 0.2, 0.5. and 0.8 are denoted as small, moderate, and large, respectively [35]. To assess changes in the ReadMI metrics between the sessions and group status, a series of mixed models were developed with group, time, and group-by-time interaction entered into the model. A linear mixed model (LMM) was used for doctor speak time and percentage of open questions. An LMM extends the linear regression model by allowing fixed and random effects [36–38]. LMMs are useful as they can account for repeated measures and can handle missing data [36–38]. Subject-specific random intercepts and slopes were used to account for correlation due to having repeated observations [38]. A generalized linear mixed model (GLMM) with a negative binomial distribution and a log link was used for the open-ended questions, closed-ended questions, total questions, reflections, scale, and ratio metrics. A GLMM was used due to the non-normal data and the repeated measures per individual [38–39]. A random intercepts model was used to account for the random variation between individuals [38]. Adjusted means were calculated and compared using a Tukey’s adjustment. All data were analyzed using SAS version 9.4 (Cary, NC), and p-values < 0.05 were regarded as statistically significant.

## Results

### Participant data

Table 2 summarizes the demographics of the medical student sample. The majority of the students were white (70%), female (67%), and had English as their primary language (91%). Additionally, the students were similar across demographics between the intervention and control groups.

**Table 2:** Demographics among READMI participants (*N* = 125)*

		Intervention	Control
	**N (%)**	**N (%)**	**N (%)**
**Age**– mean (std)	26.7 (3.0)	27.3 (3.7)	26.1 (2.0)
**Group Status**			
Intervention	63 (51.6)		
Control	59 (48.4)		
**Race**			
Asian	24 (19.7)	15 (23.4)	9 (15.3)
Black	18 (14.8)	8 (12.5)	10 (17.0)
Hispanic	5 (4.1)	3 (4.7)	2 (3.4)
White	71 (57.7)	37 (57.8)	34 (57.6)
Other	5 (4.1)	1 (1.6)	4 (6.8)
**Gender**			
Male	55 (44.7)	30 (46.9)	25 (42.4)
Female	68 (55.3)	34 (53.1)	34 (57.6)
**Native Language**			
English	111 (91.0)	59 (93.7)	52 (88.1)
Non-English	11 (9.0)	4 (6.4)	7 (11.9)

*2 participants did not provide data on demographics

### Outcome evaluation

The mean number of questions answered correctly by the medical students on the 22-item MIKT was 16.0 ± 2.8 (range = 6.0 to 22.0). The intervention group had a slightly higher average number of questions answered correctly compared to the control group (16.6 versus 15.5, difference = 1.1; effect size (es) = 0.4; t = -2.2; *p* =.03).

Table 3 presents the ReadMI metrics among the medical student participants during the first two sessions and by group status. For all students, there were decreases in the average percent of time the doctor spoke (48.2% in role-play #1 versus 41.8% in role-play #2; es = 0.6; t = 4.5; *p* <.0001) and increases in the percent of questions that were open questions (62.0% in role-play #1 and 69.0% in role-play #2; es = 0.4; t = -2.67; *p* =.008).

**Table 3:** READMI metrics among medical student participants (*N* = 125)

	Total (*n* = 125)			Intervention (*n* = 65)			Control (*n* = 60)		
	Session 1	Session 2	Effect Size	Session 1	Session 2	Effect Size	Session 1	Session 2	Effect Size
	Mean (sd)	Mean (sd)		Mean (sd)	Mean (sd)		Mean (sd)	Mean (sd)	
**Doctor Speak Time**	48.2 (11.3)^a^	41.8 (11.0)^a^	0.6	46.1 (11.2)^b^	40.1 (10.0)	0.6	50.4 (11.1)^b^	43.5 (11.8)	0.6
**Open Questions**	6.8 (3.2)^a^	8.3 (3.6)^a^	0.4	7.0 (3.1)	8.5 (4.1)	0.4	6.6 (3.3)	8.1 (3.0)	0.5
**Closed Questions**	4.3 (2.8)	3.9 (2.9)	0.1	3.4 (2.4)^b^	2.8 (2.3)^c^	0.3	5.2 (2.9)^b^	5.0 (3.1)^c^	0.1
**Total Questions**	10.9 (4.4)^a^	12.2 (4.3)^a^	0.3	10.2 (4.1)	11.3 (4.4)^c^	0.3	11.6 (4.6)	13.1 (4.0)^c^	0.3
**Reflections**	6.1 (3.5)	6.5 (3.5)	0.1	4.6 (3.5)^b^	5.0 (3.4)^c^	0.1	7.8 (2.6)^b^	8.1 (2.7)^c^	0.1
**Scale**	0.7 (0.8)	0.7 (0.7)	0	0.8 (0.9)	0.8 (0.6)	0	0.7 (0.7)	0.6 (0.7)	0.1
**Ratio of Open to Closed Questions**	2.6 (2.7)^a^	3.7 (3.6)^a^	0.3	3.2 (3.1)^b^	4.8 (4.1)^c^	0.4	1.9 (2.0)^b^	2.5 (2.5)^c^	0.3
**Ratio of Reflections to Questions**	0.6 (0.4)	0.6 (0.4)	0	0.5 (0.4)^b^	0.5 (0.4)^c^	0	0.8 (0.5)^b^	0.7 (0.3)^c^	0.3
**Percent of Open Questions**	62.0 (20.0)^a^	69.0 (20.0)^a^	0.4	68.0 (20.0)^b^	75.0 (20.0)^c^	0.4	55.0 (19.0)^b^	63.0 (17.0)^c^	0.4

^a^Denotes significant differences (*p* <.05) between sessions 1 and 2 overall

^b^Denotes significant differences between groups during session 1

^c^Denotes significant differences between groups during session 2

Note: Effect size is defined as the standardized mean difference. The absolute value was taken for each measure

For the first role-plays, there were several differences in the ReadMI metrics between the control and intervention groups, with the control group “doctors” speaking longer (50.4% versus 46.1%; es = 0.4; t = 2.1; *p* =.04), using more closed-ended questions (5.2 versus 3.4; es = 0.7; t = 3.9; *p* =.0002), and having a lower percentage of questions being open-ended (55.0% versus 68.0%; t = -3.7; es = 0.7; *p* =.0003) compared to the intervention group. For the second role-plays, the intervention group had a significantly higher percentage of questions that were open-ended (75.0% versus 63.0%; es = 0.6; t = -3.55; *p* =.0005), a lower number of closed-ended questions (2.8 versus 5.0; es = 0.8; t = 4.6; *p* <.0001), a lower total number of questions (11.3 versus 13.1; es = 0.4; t = 2.5; *p* =.02), and a higher ratio of open-ended to closed-ended questions (4.8 versus 2.5; es = 0.7; t = -3.7; *p* =.0004) compared to the control group.

There were no significant group-by-time interactions for the ReadMI metrics in the mixed models. However, for the percent of questions that were open-ended, there was an overall difference between the two groups with the intervention group having a moderately higher adjusted mean percentage compared to the control group (71.3% versus 60.5%; difference = 10.8%; es = 0.5; t = -3.61; adjusted *p* =.0005), and there was an overall increase from role-play #1 to role-play #2 (61.9% versus 69.9%; difference = -8.0%; es = 0.4; t = -4.35; adjusted *p* <.0001) (data not shown).

**Fig. 2:**
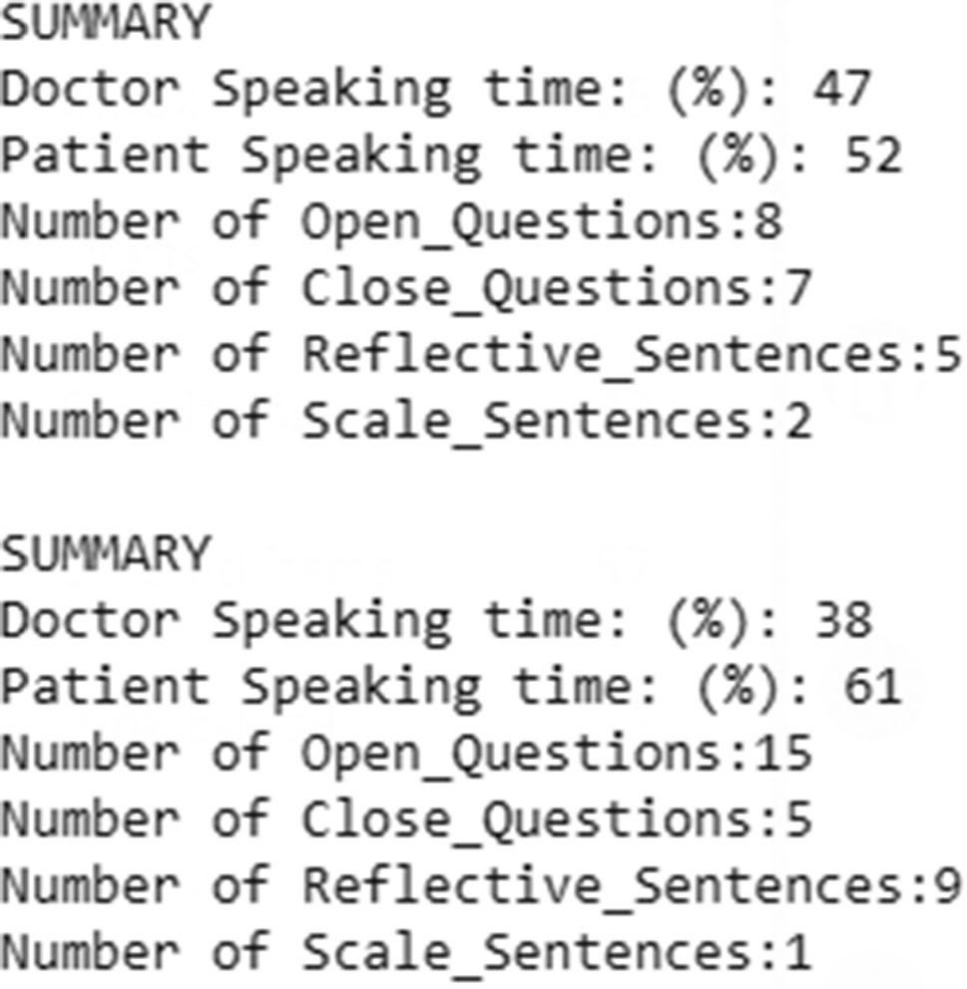
Example of ReadMI metrics for two role plays

An example of ReadMI summary data from two role-plays for the same student is shown in Fig. 2. In the intervention condition, ReadMI data for the first role-play were provided to the student after completion of that role-play, whereas in the control condition, ReadMI data for both role-plays were not shared with the students until after the completion of all role-plays.

Qualitative feedback from students suggested that the numerical data provided by ReadMI served to add credibility to the subjective feedback provided by the training facilitator. For example, a student seeing that they spoke 68% of the time during the encounter bolsters feedback regarding the need to guide the patient to do more of the problem-solving and discovery. Students indicated that they found the “ReadMI sessions” to be valuable, appreciated the quantitative as well as qualitative feedback, and generally felt that the practice increased their confidence about using the MI approach. One suggestion for improvement from a number of students was that this type of training activity occurs earlier in the medical school curriculum. No formal analyses of qualitative data comparing the control and intervention groups were done.

## Discussion

This study demonstrated that the ReadMI tool can assist in the facilitation of MI skills practice by third-year medical students with a relatively small investment of time, and that AI can be used both for the measurement of MI-relevant skills and as an instructional aid. The addition of ReadMI metrics to the feedback provided to students in the intervention group was advantageous to a statistically significant degree with respect to the physician using a moderately greater proportion of open-ended questions. In our experience, we do find that students can more readily improve their use of open questions than reflective statements.

The ReadMI tool has the dual purpose of functioning as a source of feedback in the training process by producing metrics on important communication skills, and serve as an assessment tool that can be used to quantify changes in performance. Given that there are limits on the available time for interviewing practice in most medical school curricula, use of a tool that can provide useful interviewing metrics in real time has the potential to help with time efficiency in interviewing training.

Throughout their academic careers, medical students are accustomed to receiving numbers to indicate their performance. The development and use of AI in the form of our ReadMI tool allows for real-time quantitative feedback to students, without the facilitator needing to track performance on specific communication metrics. This represents a reduction in the cognitive load on the training facilitator so that the facilitator can focus on and provide feedback about the qualitative aspects of the interview. A potential benefit of ReadMI is that it could potentially be used by students themselves for practice, without the need for faculty time.

A significant limitation of our study was the measured difference in MI skills between the intervention and control groups at baseline. This difference may have been due to the group randomization process and the timing of when students in each rotation participated. Students in the first two rotations (both randomized to the intervention condition) were closer in time to their most recent exposure to MI during the preparatory “bootcamp” prior to students beginning their clerkships, potentially contributing to the intervention groups having better baseline metrics compared to the control groups. The fact that we utilized a group rather than individual randomization process was itself a limitation of our study, a choice that was made to coincide with how motivational training is done during each clerkship rotation. Another limitation is that reliability and validity data are not available for the Motivational Interviewing Knowledge Test used in our study. Additionally, it is important to note that the intervention group received two sources of feedback after their first role play (i.e., facilitator and ReadMI metrics) whereas the control group only had one source of feedback (i.e., facilitator). The possibility cannot be ruled out that additional feedback alone is responsible for better intervention group performance rather than something specific to the ReadMI metrics. Our study sample included all third-year medical students. We did not perform a power calculation prior to the study. A limitation is that our study may not have been well-powered to assess all effects, particularly the interactions in the LMM. Finally, the fact that we did not do any formal analyses of the qualitative data comparing the intervention and control groups is a limitation of this study.

Future research of ReadMI should use more rigorous research methodology to ascertain training benefits that can be attributed to ReadMI. This will include individual versus group randomization, standardization of feedback provided by facilitators, and a “second” source of feedback for control group participants to better ensure an equal experience for all participants. Furthermore, follow-up evaluation of student performance should be done to determine whether and to what any gains in MI skills with the use of ReadMI are retained.

## Conclusion

Training in MI can be a time-consuming endeavor, both for a training facilitator and for a learner, and available time for such learning can be quite limited in medical school curricula. The use of human-AI teaming in this endeavor is a way to help make MI training more manageable and engaging for medical students. Medical school graduates who have learned to incorporate the MI approach into their interaction with patients are better equipped to facilitate the patient engagement and activation that are crucial in effective chronic disease management. Our study demonstrates that AI can be utilized to augment MI training, permitting a training facilitator to teach this important patient-centered approach effectively in a time-efficient manner.

## Data Availability

The datasets used and analyzed for this study are available from the corresponding author on reasonable request.

## Abbreviations

MI: Motivational interviewing
ReadMI: Real-time Assessment of Dialogue in Motivational Interviewing
AI: Artificial intelligence
IRB: Institutional Review Board
MIKT: Motivational Interviewing Knowledge Test
LMM: Linear mixed model
GLMM: Generalized linear mixed model
